# Extended field-of-view ultrasound imaging is reliable for measuring Transversus Abdominis muscle size at rest and during contraction

**DOI:** 10.1186/s12891-021-04157-0

**Published:** 2021-03-17

**Authors:** A. Wayne Johnson, Lauren Adams, Jade B. Kho, Daniel M. Green, Nicolas B. Pace, Ulrike H. Mitchell

**Affiliations:** Department of Exercise Sciences, Brigham Young University, 106 Smith Fieldhouse, Provo, UT 84602 USA

**Keywords:** Abdominal muscles, Measurement error, Minimal detectable difference, Panoramic ultrasound imaging, EFOV

## Abstract

**Background:**

The strength and size of core muscles, including the abdominal muscles, are crucial to proper function in most activities. Therefore, it is important to reliably assess these characteristics. Our primary objective was to determine if the length, thickness and cross-sectional area of the transversus abdominis (TrA) can be visualized independently from the internal and external abdominal oblique muscles using extended field of view ultrasound imaging at rest and with contraction and to establish its intra- and inter-tester reliability.

**Methods:**

Twenty-six individuals were recruited to participate in the study (20 F, 6 M), average age 24.0 years (SD 9.4), height 170.7 cm (SD 8.6) and weight 63.9 kg (SD 9.0). From this total number of participants, two groups of 16 randomly selected participants were assessed to determine intra- and inter-tester reliability respectively. Extended field of view ultrasound images were obtained at three vertebral levels during rest and contraction in the side lying position for both the right and left sides of the trunk.

**Results:**

Excellent intra-tester and inter-tester reliability was seen (ICC range of 0.972 to 0.984). The overall average percent standard error of the measurement for all measurements and locations was approximately 4%. The overall average minimal difference for the thickness measurement for the resting and contraction conditions combined were as follows: intratester 0.056 (0.014) cm and intertester 0.054 (0.017) cm, for area intratester 0.287 (0.086) cm^2^ and intertester 0.289 (0.101) cm^2^ and for length intratester 0.519 (0.097) cm and intertester 0.507 (0.085) cm.

**Conclusions:**

Extended field of view ultrasound imaging is an effective method of reliably capturing clear images of the TrA during rest and contraction. It provides an efficient mechanism for the analysis of muscle morphology by being able to measure the cross-sectional area, thickness, and length on one image. This methodology is recommended for studies investigating TrA function and training.

## Introduction

Diagnostic imaging, also called medical imaging, uses technologies to produce images of internal structures that cannot be seen or diagnosed otherwise. The Gold standard for visualizing and assessing muscle size is via magnetic resonance imaging (MRI) scan or computerized tomography (CT). While MRI scanning might be cost-prohibitive or not available, CT requires x-ray exposure and might therefore be contra-indicated. In addition, there are concerns that a muscle’s activation, or its ability to contract, cannot be accurately captured via MRI because of the relative long duration of each scan. Furthermore, it has been rather difficult to image larger muscles, such as the transversus abdominis (TrA) in their entirety. The TrA attaches to the costal cartilages, thoracolumbar fascia and the pelvis, wrapping around the lateral aspect of the torso [1]. Appropriate activation of the TrA is critical to proper function and for providing essential spinal stability [2]. It has also been implicated in low back pain [3]. Thus, its activation ability, as demonstrated by its size at rest and size change with contraction, has been the subject of much research, imaging efforts [4–8] and debate [9, 10].

Panoramic, or extended field-of-view ultrasound imaging (EFOV), is a technique, where the ultrasound probe is moved along an anatomical structure producing one long image that displays its anatomical context [7, 11, 12]. This technique has been reported to be reliable for simultaneous assessment of both muscle size and quality [11], valid for monitoring muscle atrophy and hypertrophy of the quadriceps [7, 13] and to have high repeatability for measuring cross-sectional area (CSA) of the abdominal muscles [7].

Tanaka et al. [7] examined the reliability of EFOV to obtain the CSA of the abdominal muscles as a group (“abdominal oblique muscles”). For this, scans were taken on two separate days. The participants were told to breathe in and, while holding their breath, the scans were taken. That study found excellent intra-tester reliability between the measurements taken from only one contraction state (breath held for 10 s) of the TrA, which is neither fully relaxed nor fully contracted. The amount of reliability likely varies between relaxed and contracted states. The muscle’s contraction ability (i.e. shortening with contraction) can be of value when assessing possible side-to-side imbalances [14]. Chen et al. [8] used EFOV to measure anterior and posterior slide of the TrA during contraction, but did not measure its change in length directly. There likely are advantages to directly measuring muscle length changes with contraction as it could be related to joint or core stability [15]. For example, researchers have shown significant TrA length shortening differences within athletic populations using magnetic resonance imaging MRI [15]. The TrA contracts best when performing a ‘hollowing maneuver’, where the navel gets actively pulled to the spine [16].

The positioning of the participant during the ultrasound scanning of the lateral abdominals ranges from supine hook-lying over sidelying [7] to 4-point kneeling [17]. While the supine position lends itself better for repeatability, it makes using the EFOV mode impossible, because the posterior insertion of the muscle cannot be reached. The 4-point kneeling position might present an alternative but could be difficult to assume for some participants. In addition, the anterior portion of the muscle could be obscured by excessive adipose tissue accumulated by the pull of gravity. This is less of a problem with the participant in the sidelying position, because the tissue is pulled away from the area of interest.

Several questions are left unanswered, such as, can the TrA be visualized independently from the other lateral abdominal muscles using EFOV? Answering this question is of value because of the TrA’s unique fiber orientation in relation to the other abdominal muscles [18] and its purported importance in core stability [19, 20], functional movement [21], and association with low back pain [6, 17, 22]. What is the intra- and inter-tester reliability? Can EFOV reliably measure changes that the muscle undergoes with contraction, namely changes in length, thickness and CSA? Thus, our objectives were to determine if the length, thickness and CSA of the TrA can be visualized independently from the internal and external oblique muscles using EFOV at rest and with contraction and to establish its intra- and inter-tester reliability, percent standard measure of the measurement and minimal detectable difference.

## Methods

### Participants

Twenty-six individuals were recruited to participate in the study (20 F, 6 M), average age 24.0 years (SD 9.4), height 170.7 cm (SD 8.6) and weight 63.9 kg (SD 9.0). From this total, two groups of 16 randomly selected participants were assessed to determine intra- and inter-tester reliability respectively. One group of 16 was used to determine intra-tester reliability while the other group of 16 was used to determine inter-tester reliability. Thus some individuals were present in both groups. Exclusion criterion was the inability to lie on the side for 20 min. Inclusion criterion was being 18 years old or older. Subjects were not excluded if they had low back pain. Prior to inclusion in the study, all participants acknowledged their willingness to participate in the study by reading and asking questions about the study informed consent. Once satisfied and having agreed to participate they signed the informed consent. This study received appropriate human subject approval from the Human Research Protection Program and Institutional Review Board at Brigham Young University and was assigned study number, X2019–336. This study was conducted in accordance with the relevant guidelines and regulations to meet appropriate safeguards to protect the rights and welfare of research subjects in compliance with the Declaration of Helsinki and Federal regulations 45 CFR 46.111.

### Ultrasound imaging

**Fig. 1:**
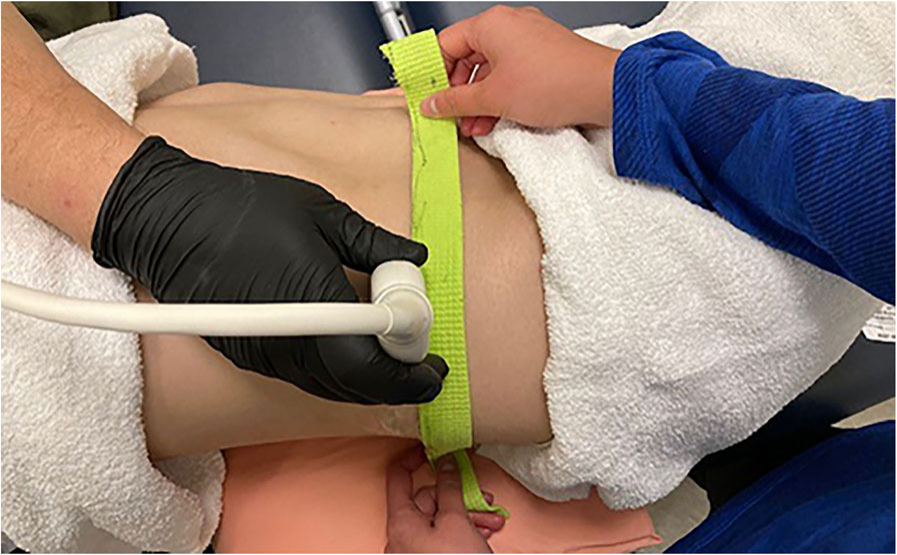
Extended field of view ultrasound imaging of the transversus abdominis muscle in side-lying with guide strip to maintain appropriate spinal level

Each image was taken with the subject in side-lying position. A pillow was placed into the area between the table and trunk to help reduce the lateral curvature of the spine and maintain this position. A researcher palpated the participant’s spinal column to locate and mark spinal levels L1, L3 and L5. A flexible strip of 4 mm thick foam was held in place by a researcher on the participant’s side and used as a guide to ensure the probe remained parallel to the spinal level during imaging (Fig. 1). The posterior aspect of the TrA at its attachment to the thoracolumbar fascia was visualized. With the probe in transverse orientation, the TrA muscle was scanned by gliding the soundhead anteriorly along the foam strip until the anterior aspect was fully visualized. We used LogicView mode (GE Logic S8 6–15 MHz probe, GE Healthcare Chicago, IL) to obtain a panoramic image of the TrA muscle. Imaging order for right and left sides was randomized, while spinal levels were consistently measured from distal (L5) to proximal (L1). The muscle was imaged twice on each subject at each of the three spinal levels (L5, L3 and L1) during relaxed and contracted state, yielding 12 separate images on each side for a total of 24 images per person. These three levels were chosen to provide a more complete impression of TrA by utilizing the available imaging window between the lower ribs and iliac crest. The contraction was performed via the ‘abdominal hollowing’ procedure, which has been shown to strongly contract the TrA muscle [16]. The participant was trained in the hollowing maneuver prior to image collection to ensure correct performance. After completing imaging of the first side, the participant was asked to lie on the other side, to allow imaging of the contralateral TrA. The spinal column was palpated and marked anew to assure measurement occurred at proper levels. The entire process was performed again as described above.

### Data processing/measuring protocol/image processing

**Fig. 2:**
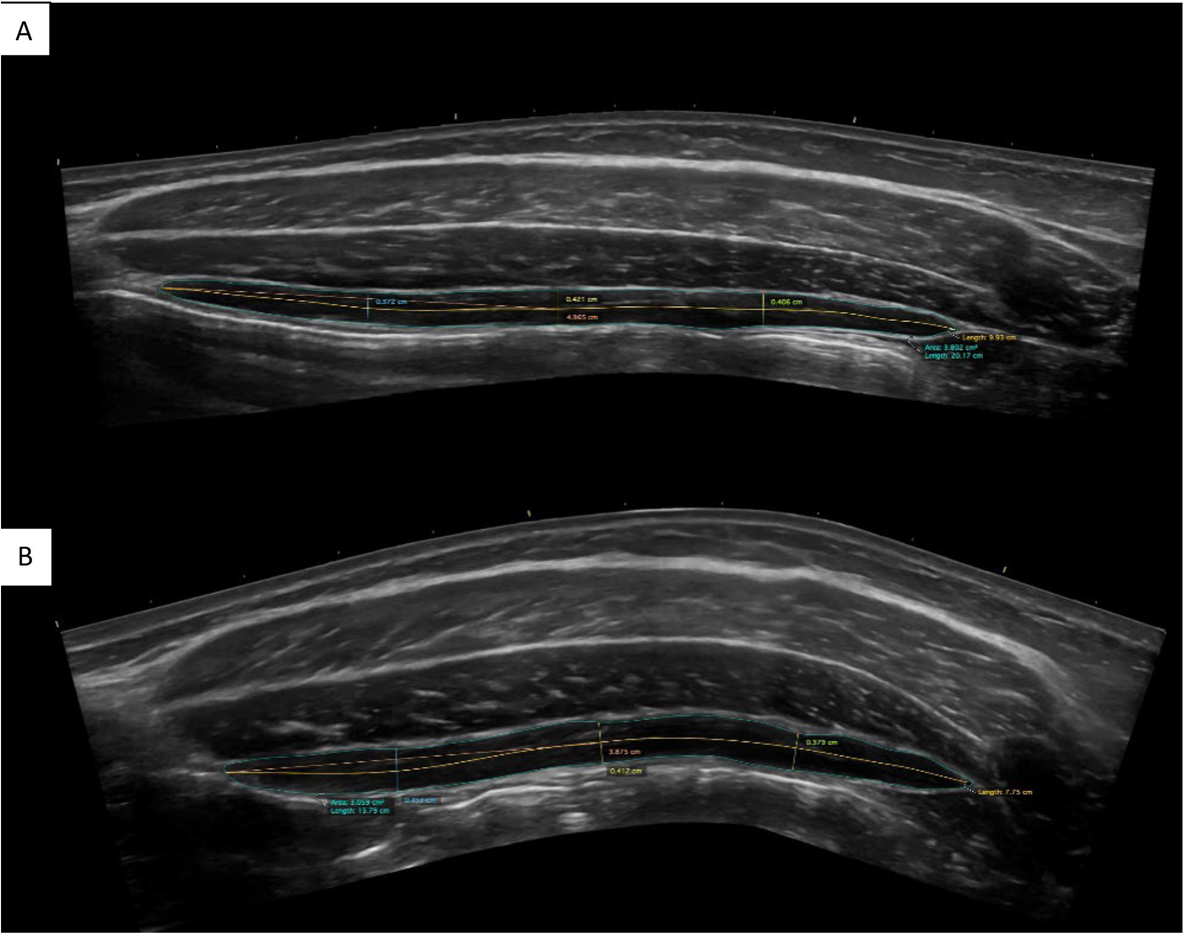
Extended field of view measurement of transversus abdominis muscle thickness, length, and cross-sectional area at rest (**a**) and during contraction (**b**)

The ultrasound images were measured at a later time following the data collection. The researchers received two months of intensive training and practice prior to the start of the study in both scanning and image processing. Specifically, two researchers were randomly assigned to capture the images. The postprocessing measurements were conducted using the Osirix DICOM Viewer (Pixmeo SARL, 266 Rue de Bernex, CH-1233 Bernex, Switzerland). Each subject’s 24 US scans were measured twice by two separate randomly assigned researchers to obtain the thickness, area, and length. See Fig. 2. The CSA was measured using the closed polygon function to trace along the internal edge of the fascial borders of the muscle. The length measurement was performed using the open polygon function through the center of the muscle from the anterior to posterior myofascial junctions, while staying equidistant from the superficial and deep fascial borders in order to create a line that followed the specific contour of the muscle. The average thickness measurement was obtained by measuring three separate locations using the straight-line function. These occurred at the 25, 50 and 75% points along the measured length of the TrA. An average of these three measurements was then taken and reported as the overall thickness.

#### Data/statistical analysis

For our intra-tester and inter-tester reliability assessments, we calculated the Intraclass Correlation Coefficients (ICC) model ICC_(3,k)_ with random subjects and fixed operators for absolute agreement. The assessed study participants were randomly chosen from a larger data collection and thus varied between the intratester and intertester analyses. ICC values of 0.6 to 0.8 were considered moderate, while ICC between 0.8 and 0.9 were considered good, and ICC above 0.9 were considered excellent [23]. The 95% confidence intervals for the ICCs were also calculated. In order to further understand these reliability measurements, the Standard Error of the Measurement (SEM) and minimal difference (MD; MD = SEM*1.96*Square root of 2), which is the minimal detectable change to be considered real, were calculated [24]. ANOVA was conducted to analyze differences between right and left TrA size between the three levels. We used SPSS statistical software (IBM Corp. IMB SPSS Statistics, version 26.0. Armonk, NY: IBM Corp) with an alpha set at 0.05.

## Results

Mean and standard deviation values for the measurement of the TrA at the 3 levels on the right and left trunk at rest and during hollowing along with Intraclass correlation values, Standard Error of the Measurement, Percent SEM, minimal difference values, average ICC, SEM, %SEM and MD values are in Tables 1, 2, 3, 4, 5 and 6.

**Table 1:** Intratester Reliability of the measurement of Thickness of the Transverse Abdominis muscle at three Lumbar levels at rest and during the hollowing maneuver

Resting										
	Right Mean (SD) cm	Left Mean (SD) cm	ICC (95% CI)		SEM (cm) (95% CI for muscle size)		% SEM		Minimal Difference (cm)	
**Level**			**Right**	**Left**	**Right**	**Left**	**Right**	**Left**	**Right**	**Left**
**L1**	**0.394 (0.096)**	**0.404 (0.099)**	**0.985** **(0.967–0.994)**	**0.976** **(0.947–0.991)**	**0.012** **(0.37–0.42)**	**0.015** **(0.374–0.434)**	**3.046**	**3.713**	**0.033**	**0.043**
**L3**	**0.370 (0.078)**	**0.393 (0.108)**	**0.971** **(0.936–.989)**	**0.969** **(0.933–0.988)**	**0.016** **(0.339–0.403)**	**0.019** **(0.355–0.430)**	**3.846**	**4.835**	**0.044**	**0.053**
**L5**	**0.355 (0.071)**	**0.340 (0.091)**	**0.926** **(0.838–0.972)**	**0.976** **(0.947–0.991)**	**0.019** **(0.32–0.39)**	**0.014** **(0.311–0.367)**	**5.352**	**4.118**	**0.053**	**0.039**
**Average value (SD)**	**0.376 (0.09)**		**0.97 (0.02)**		**0.02 (0.003)**		**4.15 (0.83)**		**0.04 (0.008)**	
**Hollowing**										
	**Right Mean (SD) cm**	**Left Mean (SD) cm**	**ICC (95% CI)**		**SEM (95% CI for muscle size)**		**%SEM**		**Minimal Difference (cm)**	
**Level**			**Right**	**Left**	**Right**	**Left**	**Right**	**Left**	**Right**	**Left**
**L1**	**0.541 (0.153)**	**0.567 (0.148)**	**0.972** **(0.938–0.989)**	**0.973** **(0.942–0.990)**	**0.025** **(0.49–0.59)**	**0.024** **(0.519–0.615)**	**4.621**	**4.233**	**0.071**	**0.067**
**L3**	**0.523 (0.162)**	**0.561 (0.157)**	**0.976** **(0.947–0.991)**	**0.977** **(0.951–0.992)**	**0.025** **(0.48–0.58)**	**0.024** **(0.515–0.610)**	**4.780**	**4.278**	**0.069**	**0.066**
**L5**	**0.494 (0.186)**	**0.545 (0.246)**	**0.977** **(0.949–0.991)**	**0.992** **(0.983–0.997)**	**0.028** **(0.44–0.55)**	**0.022** **(0.502–0.588)**	**5.668**	**4.037**	**0.078**	**0.061**
**Average value (SD)**	**0.539 (0.18)**		**0.98 (0.01)**		**0.02 (0.002)**		**4.60 (0.59)**		**0.07 (0.006)**

**Table 2:** Intratester Reliability of the measurement of Area of the Transverse Abdominis muscle at three Lumbar levels at rest and during the hollowing maneuver

Resting										
	Right Mean (SD) cm^**2**^	Left Mean (SD) cm^**2**^	ICC (95% CI)		SEM (95% CI for muscle size)		% SEM		Minimal Difference (cm^**2**^)	
**Level**			**Right**	**Left**	**Right**	**Left**	**Right**	**Left**	**Right**	**Left**
**L1**	**2.51 (0.68)**	**2.76 (0.88)**	**0.980** **(0.956–0.992)**	**0.979** **(0.953–0.993)**	**0.096** **(2.32, 2.70)**	**0.127** **(2.51, 3.01)**	**3.825**	**4.601**	**0.265**	**0.353**
**L3**	**2.27 (0.64)**	**2.46 (0.79)**	**0.965** **(0.921–0.987)**	**0.990** **(0.979–0.996)**	**0.119** **(2.04, 2.50)**	**0.079** **(2.31, 2.62)**	**5.242**	**3.211**	**0.330**	**0.220**
**L5**	**1.97 (0.42)**	**2.04 (0.76)**	**0.962** **(0.916–0.986)**	**0.985** **(0.967–0.994)**	**0.083** **(1.80, 2.13)**	**0.094** **(1.86, 2.23)**	**4.213**	**4.608**	**0.230**	**0.260**
**Average value (SD)**	**2.34 (0.70)**		**0.98 (0.01)**		**0.10 (0.02)**		**4.28 (0.71)**		**0.28 (0.05)**	
**Hollowing**										
	**Right Mean (SD) cm**^**2**^	**Left Mean (SD) cm**^**2**^	**ICC (95% CI)**		**SEM (95% CI for muscle size)**		**%SEM**		**Minimal Difference (cm**^**2**^**)**	
**Level**			**Right**	**Left**	**Right**	**Left**	**Right**	**Left**	**Right**	**Left**
**L1**	**2.46 (0.72)**	**3.02 (1.15)**	**0.978** **(2.33–2.54)**	**0.977** **(0.950–0.991)**	**0.106** **(2.25, 2.66)**	**0.175** **(2.68, 3.36)**	**4.711**	**5.795**	**0.295**	**0.484**
**L3**	**2.32 (0.58)**	**2.49 (0.91)**	**0.970** **(0.933–0.989)**	**0.993** **(0.984–0.997)**	**0.101** **(2.12, 2.52)**	**0.076** **(2.34, 2.64)**	**4.353**	**3.057**	**0.281**	**0.211**
**L5**	**1.84 (0.60)**	**2.36 (1.22)**	**0.991** **(0.980–0.997)**	**0.989** **(0.977–0.996)**	**0.057** **(1.73, 1.96)**	**0.128** **(2.11, 2.61)**	**3.098**	**5.424**	**0.158**	**0.355**
**Average value (SD)**	**2.41 (0.86)**		**0.98 (0.01)**		**0.11 (0.04)**		**4.41 (1.50)**		**0.30 (0.11)**

**Table 3:** Intratester Reliability of the measurement of Length of the Transverse Abdominis muscle at three Lumbar levels at rest and during the hollowing maneuver

Resting										
	Right Mean (SD) cm	Left Mean (SD) cm	Resting ICC (95% CI)		Resting SEM (95% CI for muscle size)		Resting % SEM		Resting Minimal Difference (cm)	
**Level**			**Right**	**Left**	**Right**	**Left**	**Right**	**Left**	**Right**	**Left**
**L1**	**6.78 (1.61)**	**7.31 (1.30)**	**0.990** **(0.978–0.996)**	**0.986** **(0.970–0.995)**	**0.161** **(6.47, 7.10)**	**0.154** **(7.00, 7.61)**	**2.376**	**2.107**	**0.447**	**0.427**
**L3**	**6.52 (1.69)**	**6.66 (1.63)**	**0.982** **(0.960–0.993)**	**0.978** **(0.952–0.992)**	**0.228** **(6.07, 6.96)**	**0.241** **(6.19, 7.13)**	**3.50**	**3.619**	**0.631**	**0.669**
**L5**	**6.08 (1.60)**	**6.37 (1.41)**	**0.988** **(0.974–0.996)**	**0.970** **(0.932–0.989)**	**0.175** **(5.73, 6.42)**	**0.244** **(6.19, 7.13)**	**2.878**	**3.830**	**0.486**	**0.677**
**Average value (SD)**	**6.62 (1.54)**		**0.98 (0.01)**		**0.20 (0.042)**		**3.05 (0.71)**		**0.56 (0.12)**	
**Hollowing**										
	**Right Mean (SD) cm**	**Left Mean (SD) cm**	**ICC (95% CI)**		**SEM (95% CI for muscle size)**		**%SEM**		**Minimal Difference (cm)**	
**Level**			**Right**	**Left**	**Right**	**Left**	**Right**	**Left**	**Right**	**Left**
**L1**	**5.18 (1.44)**	**5.93 (1.50)**	**0.990** **(0.978–0.996)**	**0.980** **(0.956–0.992)**	**0.144** **(4.90, 5.46)**	**0.211** **(5.51, 6.34)**	**2.939**	**3.558**	**0.398**	**0.586**
**L3**	**5.01 (1.24)**	**4.88 (1.09)**	**0.976** **(0.947–0.991)**	**0.971** **(0.936–0.989)**	**0.173** **(4.67, 5.35)**	**0.185** **(4.52, 5.25)**	**3.45**	**3.791**	**0.480**	**0.514**
**L5**	**4.36 (1.35)**	**4.50 (1.03)**	**0.983** **(0.963–0.994)**	**0.977** **(0.949–0.991)**	**0.175** **(4.02, 4.71)**	**0.157** **(4.20, 4.81)**	**4.014**	**3.489**	**0.486**	**0.435**
**Average value (SD)**	**4.98 (1.28)**		**0.98 (0.01)**		**0.17 (0.023)**		**3.54 (0.36)**		**0.48 (0. 06)**

**Table 4:** Intertester Reliability of the measurement of Thickness of the Transverse Abdominis muscle at three Lumbar levels at rest and during the hollowing maneuver

Resting										
	Right Mean (SD) cm	Left Mean (SD) cm	ICC (95% CI)		SEM (95% CI for muscle size)		% SEM		Minimal Difference (cm)	
**Level**			**Right**	**Left**	**Right**	**Left**	**Right**	**Left**	**Right**	**Left**
**L1**	**0.390 (0.092)**	**0.384 (0.098)**	**0.990** **(0.983–0.997)**	**0.956** **(0.907–0.983)**	**0.028** **(0.34–0.45)**	**0.021** **(0.384–0.424)**	**2.48**	**5.340**	**0.027**	**0.057**
**L3**	**0.370 (0.075)**	**0.356 (0.103)**	**0.975** **(0.947–.990)**	**0.972** **(0.940–0.989)**	**0.012** **(0.347–0.393)**	**0.017** **(0.323–0.390)**	**3.21**	**4.850**	**0.033**	**0.048**
**L5**	**0.351 (0.068)**	**0.321 (0.097)**	**0.942** **(0.876–0.977)**	**0.973** **(0.943–0.990)**	**0.016** **(0.32–0.38)**	**0.016** **(0.290–0.353)**	**4.68**	**4.943**	**0.045**	**0.044**
**Average value (SD)**	**0.362 (0.09)**		**0.97 (0.02)**		**0.02 (0.006)**		**4.25 (1.13)**		**0.04 (0.011)**	
**Hollowing**										
	**Right Mean (SD) cm**	**Left Mean (SD) cm**	**ICC (95% CI)**		**SEM (95% CI for muscle size)**		**%SEM**		**Minimal Difference (cm)**	
**Level**			**Right**	**Left**	**Right**	**Left**	**Right**	**Left**	**Right**	**Left**
**L1**	**0.535 (0.150)**	**0.544 (0.149)**	**0.961** **(0.918–0.985)**	**0.980** **(0.958–0.992)**	**0.030** **(0.478–0.593)**	**0.021** **(0.503–0.585)**	**5.535**	**3.866**	**0.082**	**0.054**
**L3**	**0.515 (0.153)**	**0.532 (0.148)**	**0.985** **(0.967–0.994)**	**0.972** **(0.940–0.989)**	**0.019** **(0.479–0.552)**	**0.025** **(0.483–0.610)**	**3.626**	**4.672**	**0.052**	**0.069**
**L5**	**0.492 (0.182)**	**0.493 (0.164)**	**0.974** **(0.944–0.990)**	**0.986** **(0.971–0.995)**	**0.029** **(0.434–0.549)**	**0.019** **(0.455–0.531)**	**5.962**	**3.935**	**0.081**	**0.058**
**Average value (SD)**	**0.52 (0.16)**		**0.98 (0.01)**		**0.02 (0.005)**		**4.60 (0.97)**		**0.07 (0.013)**

**Table 5:** Intertester Reliability of the measurement of Area of the Transverse Abdominis muscle at three Lumbar levels at rest and during the hollowing maneuver

Resting										
	Right Mean (SD) cm^**2**^	Left Mean (SD) cm^**2**^	ICC (95% CI)		SEM (95% CI for muscle size)		% SEM		Minimal Difference (cm^**2**^)	
**Level**			**Right**	**Left**	**Right**	**Left**	**Right**	**Left**	**Right**	**Left**
**L1**	**2.41 (0.66)**	**2.63 (0.97)**	**0.975** **(0.948–0.990)**	**0.987** **(0.973–0.995)**	**0.105** **(2.21, 2.62)**	**0.110** **(2.41, 2.84)**	**4.34**	**4.21**	**0.291**	**0.306**
**L3**	**2.12 (0.60)**	**2.16 (0.72)**	**0.982** **(0.961–0.993)**	**0.984** **(0.966–0.994)**	**0.081** **(1.96, 2.28)**	**0.091** **(1.98, 2.34)**	**3.80**	**4.23**	**0.223**	**0.253**
**L5**	**1.77 (0.45)**	**1.77 (0.66)**	**0.977** **(0.951–0.991)**	**0.989** **(0.976–0.996)**	**0.069** **(1.63, 1.90)**	**0.070** **(1.63, 1.90)**	**3.89**	**3.94**	**0.191**	**0.193**
**Average value (SD)**	**2.14 (0.67)**		**0.98 (0.01)**		**0.09 (0.017)**		**4.07 (0.22)**		**0.24 (0.049)**	
**Hollowing**										
	**Right Mean (SD) cm**^**2**^	**Left Mean (SD) cm**^**2**^	**ICC (95% CI)**		**SEM (95% CI for muscle size)**		**%SEM**		**Minimal Difference (cm**^**2**^**)**	
**Level**			**Right**	**Left**	**Right**	**Left**	**Right**	**Left**	**Right**	**Left**
**L1**	**2.48 (0.92)**	**2.83 (1.25)**	**0.972** **(0.940–0.989)**	**0.980** **(0.958–0.992)**	**0.153** **(2.18, 2.79)**	**0.177** **(2.49, 3.18)**	**6.19**	**6.25**	**0.425**	**0.491**
**L3**	**2.10 (0.56)**	**2.40 (1.27)**	**0.972** **(0.940–0.989)**	**0.988** **(0.974–0.995)**	**0.093** **(1.92, 2.28)**	**0.139** **(2.13, 2.68)**	**4.45**	**5.77**	**0.259**	**0.384**
**L5**	**1.74 (0.58)**	**2.02 (1.01)**	**0.991** **(0.980–0.996)**	**0.989** **(0.977–0.996)**	**0.055** **(1.63, 1.85)**	**0.201** **(1.58, 2.37)**	**3.17**	**5.26**	**0.153**	**0.294**
**Average value (SD)**	**2.26 (0.74)**		**0.98 (0.01)**		**0.14 (0.054)**		**5.18 (1.19)**		**0.33 (0.123)**

**Table 6:** Intertester Reliability of the measurement of Length of the Transverse Abdominis muscle at three Lumbar levels at rest and during the hollowing maneuver

Resting										
	Right Mean (SD) cm	Left Mean (SD) cm	ICC (95% CI)		SEM (95% CI for muscle size)		% SEM		Minimal Difference (cm)	
**Level**			**Right**	**Left**	**Right**	**Left**	**Right**	**Left**	**Right**	**Left**
**L1**	**6.7 (1.57)**	**7.21 (2.00)**	**0.981** **(0.958–0.992)**	**0.992** **(0.973–0.995)**	**0.216** **(6.27, 7.12)**	**0.178** **(6.86, 7.56)**	**3.22**	**2.47**	**0.598**	**0.494**
**L3**	**6.18 (1.53)**	**6.41 (1.23)**	**0.991** **(0.981–0.996)**	**0.981** **(0.960–0.993)**	**0.145** **(5.89, 6.46)**	**0.170** **(6.07, 6.74)**	**2.34**	**2.66**	**0.401**	**0.472**
**L5**	**5.60 (1.68)**	**5.89 (1.30)**	**0.991** **(0.981–0.997)**	**0.981** **(0.960–0.993)**	**0.160** **(5.28, 5.91)**	**0.180** **(5.54, 6.24)**	**2.85**	**3.05**	**0.443**	**0.497**
**Average value (SD)**	**6.33 (1.55)**		**0.99 (0.01)**		**0.17 (0.024)**		**2.77 (0.339)**		**0.48 (0.066)**	
**Hollowing**										
	**Right Mean (SD) cm**	**Left Mean (SD) cm**	**ICC (95% CI)**		**SEM (95% CI for muscle size)**		**%SEM**		**Minimal Difference (cm)**	
**Level**			**Right**	**Left**	**Right**	**Left**	**Right**	**Left**	**Right**	**Left**
**L1**	**5.14 (1.45)**	**5.73 (1.69)**	**0.979** **(0.956–0.992)**	**0.982** **(0.961–0.993)**	**0.210** **(4.73, 5.55)**	**0.226** **(5.29, 6.17)**	**4.09**	**3.95**	**0.583**	**0.627**
**L3**	**4.73 (1.30)**	**4.82 (1.34)**	**0.975** **(0.942–0.990)**	**0.975** **(0.948–0.990)**	**0.205** **(4.33, 5.13)**	**0.212** **(4.41, 5.24)**	**4.35**	**4.40**	**0.570**	**0.587**
**L5**	**4.18 (1.45)**	**4.29 (1.14)**	**0.987** **(0.973–0.995)**	**0.987** **(0.973–0.995)**	**0.165** **(3.85, 4.50)**	**0.130** **(4.04, 4.55)**	**3.95**	**3.02**	**0.457**	**0.360**
**Average value (SD)**	**4.82 (1.40)**		**0.98 (0.01)**		**0.04 (0.036)**		**3.96 (0.50)**		**0.53 (0.101)**	

### Intratester comparison

We observed excellent intratester repeatability in assessing TrA thickness, length, and area at rest and during contraction on both sides and across all three levels, Tables 1, 2 and 3. The average ICC values ranged from 0.973 to 0.980. The average overall SEM ranged from 0.020 (0.005) cm for thickness to 0.187 (0.035) cm for length. The overall average percent SEM for all measurements and locations was 4.006% (0.590), meaning that when performing any of the measurements one could expect to have on average 4% error in the measurement. The specific average percent SEM measurements showed higher values for thickness (4.377 (0.723) %), followed by area (4.345 (0.911) %), while length had the lowest average percent SEM (3.296 (0.594) %). The average MD for the measurement of thickness with rest and contracted conditions combined were 0.056 (0.014) cm, for area 0.287 (0.086) cm^2^, and for length 0.519 (0.097) cm. The MDs were generally smaller for the rest conditions (thickness 0.044 (0.008) cm; area 0.276 (0.054) cm^2^; length 0.556 (0.115) cm, compared to the contracted conditions (thickness 0.069 (0.006) cm; area 0.297 (0.114) cm^2^, except for the length measurement (0.483 (0.065) cm).

### Intertester comparison

We observed a similar excellent intertester reliability in assessing TrA thickness, length, and area at rest and during contraction across all three levels and both sides of the abdomen, Tables 4, 5 and 6. The average ICC value ranged from 0.972 to 0.984. The average overall SEM was 0.021 (0.006) cm for thickness measurements, 0.136 (0.054) cm^2^ for area and 0.183 (0.031) cm for length. The overall percent SEM for all measurements and locations was similar to the intratester comparison with a value of 4.14 (1.06). Again, this indicates about a 4% error rate for measurements of TRA muscle size. The specific percent SEM measurements showed higher percent values for area (5.181 (1.191) %) followed by thickness (4.424 (1.021) %), while length again had the smallest average percent SEM (3.362 (0.745) %). The overall average minimal difference for the measurement for thickness for both conditions combined was 0.054 (0.017) cm, for area 0.289 (0.101) cm^2^, and for length 0.507 (0.085) cm. The average minimal differences were all smaller for the rest condition (thickness: 0.042 (0.011) cm; area: 0.243 (0.049) cm^2^; length: 0.484 (0.066) cm compared to the contracted condition (thickness 0.066 (0.013) cm; area 0.334 (0.123) cm^2^; length 0.531 (0.101) cm).

## Discussion

The primary purpose of this study was to establish a reliable methodology for simultaneously imaging and measuring the length, thickness and CSA of the TrA using EFOV. The results demonstrate that our methodology is able to isolate the TrA from the other lateral oblique abdominal muscles and produce excellent reliability in the assessment of length, thickness and CSA. As far as we know, this is the first study to report a methodology for directly obtaining simultaneously these three single planar measurements of the TrA in isolation using EFOV at rest and during contraction. The TrA, as expected, progressively decreased from the largest size at L1 to the smallest at L5, except for thickness with L1 and L3 being similar in size. We noted a general symmetry in thickness and length between right and left sides in both relaxed and contracted states of the TrA. However, we noted that the overall left TrA CSA was significant larger than the right side. Despite the computational relationship between thickness and length to area, the observed difference could be due to non-significant segmental variations within thickness and length that when combined resulted in the overall difference in area. Other expected relationships were also supported in our results. For example, the TrA length was greatest at rest and smallest with contraction, while the muscle thickness was greatest with contraction compared to the resting condition.

Another purpose was to establish our method’s intra-tester and inter-tester reliability. We found excellent intra- and intertester reliability in all of the measurements performed with the muscle at rest and during contraction. Our ICC scores were similar to, or higher than those reported by other researchers using ultrasound imaging for measurements of abdominal muscle sizes with scores that ranged from 0.84 to 0.99 [7, 17, 22, 25]. Those ICC scores indicate good to excellent reliability. It is of note that those papers assessed intra-tester reliability, while we also assessed inter-tester reliability. In addition, most of them only assessed muscle thickness [5, 6, 17, 25–27], except for Tanaka et al. [7], who measured area of the three combined lateral abdominal muscles and Chen et al. [8], who measured TrA length at rest and anterior/posterior slide, not the change in length with contraction. These latter studies are of particular interest to the current study as they are the only other studies using EFOV to assess the lateral abdominal muscles. Tanaka et al. [7] reported excellent reliability with ICC values of 0.955 to 0.958 while Chen et al. [8] reported good to excellent reliability with ICC range of 0.888 to 0.978. EFOV has been used to measure muscle size in other parts of the body and was also found to produce good to excellent reliability, e.g., medial gastrocnemius with an ICC of 0.914 [11], the quadriceps with an ICC range of 0.78 to 0.986 [13, 28], and hamstrings with ICC values ranging from 0.71 to 0.99 [28].

In addition to high ICC scores, a good measurement tool should also have low error rates, as indicated in low SEMs and MDs. Our SEMs and MDs were comparable to, and even lower than those seen in other studies. For example, our TrA thickness SEM is within the range of SEM reported in other studies (0.01 to 0.023 cm [25, 27]. Only one other study [7] presented SEMs and MDs with their EFOV data when imaging abdominal muscles, however that study grouped the three lateral abdominal muscles into one. The percent SEM of abdominal muscle size reported by Tanaka et al. [7] was 7.3% for the area of the grouped three lateral abdominal muscles, which was higher than those found in our study (ranging from 2.8 to 5.2%). Tanaka et al. [7] report MD for these muscles of 5.3 cm^2^ or 20.3%. Our MD values range from 0.24 to 0.33 cm^2^ or 11 to 15%, comparing favorably with their values. A study assessing SEM and MD for EFOV of the medial gastrocnemius muscle area found a SEM of 0.72 cm^2^ or 5.83% SEM and a MD of 1.995 cm^2^ or 16% [11]. Again, our single muscle measurements compare well to those values. No other studies have reported SEMs for length measurements, and thus we cannot make any direct comparisons. It has been suggested that, based on its high ICC scores, low SEM and MD and its excellent agreement with MRI-based measurement (Lin’s Concordance correlation coefficient = 0.78), EFOV is a valid tool for monitoring quadriceps muscle atrophy and hypertrophy and for detecting gastrocnemius atrophy [13]. Based on our similar high reliability and low SEM and MD results, we suggest that our method of assessing TrA length, CSA and thickness provides viable and reliable measurements with low error rates. It would, however, be valuable to compare individual abdominal muscles size measurements from images obtained via MRI to those obtained with EFOV.

It is interesting to note differences in the average percent SEMs for intratester and intertester measurements. Specifically, the average percent SEM for intratester measurements was slightly higher for thickness, followed by area, while it was slightly higher for the area, followed by thickness in the intertester measurements. The variation between these could be the result of using a different subset of participants in the two analyses. It could also be a systematic error introduced by the random assignment of the researchers performing the measurements, meaning that some of researchers contributed more to the processing of one analysis than the other. However, since the length measurements showed the lowest and similar SEM in both analyses we believe that the former explanation is more likely.

Our measurement values for TrA muscle size and changes in size during contraction are reflective of values found in previous studies [4–6, 17, 25–27] and help establish a degree of validity, even though these measurements have not been compared directly to MRI. Most of the previous TrA size assessments involved thickness, while only one paper [8] assessed resting length and muscle slide with contraction. No other paper has assessed changes in TrA CSA coinciding with muscle contraction. Specifically, our average resting TrA thickness of 0.369 cm is very similar to the average value measured by Puentedura et al. [5], which was 0.30 cm (SD 0.093). Other researchers reported average values as high as 0.69 cm (SD 0.16) [4]. Similarly, our average TrA thickness during contraction (hollowing) was 0.530 cm, similar to the measurements by Puentedura (2011) [5], which was 0.55 cm (SD 0.17). Other researchers [4] reported average values as high as 1.02 cm (SD 0.18). In terms of length measurements, Chen et al. [8] found a length change of 1.87 cm in asymptomatic individuals TrA during a contraction. Our observed average overall change (1.6 cm) is within an acceptable range considering protocol differences.

Our resting thickness and overall average change in TrA thickness with contraction correspond well with values found by several other authors who used US to measure TrA thickness changes e.g. [4–6, 17, 25–27]. Our average change of TrA thickness with contraction was 0.161 cm, which is similar to the values found by Dafkou et al. [26] of 0.169, but lower than the one found by Puentedura [27], which was 0.39 cm. Their measurements of thickness using US were generally taken at one lumbar level, lateral to the subluminaris, while our measurements are averages across the length of the muscle at three specified points. There is no standardized location for the visualization and measurement of the TrA, although most papers report positioning the US probe just proximal to the iliac crest. Thus, our method of thickness measurement may provide a better overall assessment of TrA thickness, because it represents an average across the whole muscle and over multiple spinal levels.

Our overall average CSA changed 4.5% from rest to contraction (at rest 2.24cm^2^, with contraction 2.34cm^2^). We are not able to compare this change in CSA to any other studies.

There are potential limitations to this study that should be considered when evaluating the findings. There may have been variability in the amount of TrA contraction between trials, as we did not directly measure the force production during of the hollowing maneuver. We verbally encouraged a ‘rigorous’ contraction in an attempt to standardize this part of the methods. Fatigue from holding repeated contractions could have also influenced our results. We allowed individuals to rest between trials. Additionally, we also randomized the order of assessment to reduce an order effect in the measurements and to account for fatigue. The length of the torso between subjects sometimes presented a challenge. When the subject had a shorter torso (space between the iliac crest and bottom of the rib cage) the relatively lower positioned ribs tended to interfere with the prescribed imaging path of the probe, necessitating an adjustment inferiorly.

## Conclusion

Extended field of view ultrasound imaging is an effective method of reliably capturing clear images of the TrA during rest and contraction. It provides an efficient mechanism for the analysis of TrA morphology by being able to measure the cross-sectional area, thickness, and length on one image. This methodology is recommended for studies investigating TrA function and training.

## Data Availability

The datasets generated and/or analysed during the current study are not publicly available as approval was not granted by the ethical review board nor was a statement included in the informed consent that study data would be deposited in a research depository. However, researchers seeking access to de-identified data should make a reasonable request to the corresponding author and an appeal to the Human Research Protection Program and Institutional Review Board can be sought.
